# Anatomy of the Relational Reframe in Attachment‐Based Family Therapy

**DOI:** 10.1111/famp.70155

**Published:** 2026-05-14

**Authors:** Tara Santens, Chen Chu‐Chun, Suzanne Levy, Guy Diamond, Guy Bosmans

**Affiliations:** ^1^ Clinical Psychology Research Group KU Leuven Leuven Belgium; ^2^ ABFT International Training Institute Philadelphia Pennsylvania USA; ^3^ Perlman School of Medicine University of Pennsylvania Philadelphia Pennsylvania USA

**Keywords:** ABFT, attachment, family therapy, process research, relational reframing

## Abstract

In attachment‐based family therapy (ABFT), shifting the treatment goal from the adolescent as the problem to strengthening family relationships as the solution is the critical first task. No research has explored whether this “relational reframe” works, how it works, and for whom it works. We evaluated the relational reframe for 47 families receiving ABFT for depressed and suicidal adolescents. We coded markers to identify the reframing and contracting phases of the reframe intervention. We coded two key process elements: content and affect. We evaluated whether the degree to which family members discussed the themes of the reframe phase (relational ruptures and softer emotions) were associated with acceptance of the contracting goal. We explored if attachment style and self‐reported parental depression and adolescent‐reported family conflict were associated with accepting the contract for a relational focused therapy. Markers for the beginning of the reframe and contracting phase could be reliably identified. The degree to which adolescents and parents engaged in discussion about the reframe themes was associated with parents' acceptance of the contracting goal. Adolescents' dismissive attachment style was not associated with engagement in the reframe themes, but was associated with adolescents' reduced acceptance of the contract goal. Adolescents' preoccupied attachment style was associated with greater engagement in the reframing themes, but not associated with acceptance of the contract goals. Parental depression and adolescent‐ reported family conflict were not linked with acceptance of the relational reframe. This study should help therapist understand specific strategies for creating a relational repair frame for therapy and encourage researchers to study the subtle processes involved in effective therapy.

## 1 Introduction

The first challenge for many family therapists, regardless of the model they use, is to help parents and youth agree to participate in family (vs. individual) therapy. Frequently, parents call for an appointment and say, “I will drop my son at 3:00 and pick him up at 4:00.” Parents often think the adolescent is the problem, and adolescents often do not want to be in therapy at all and certainly not with their parents. So how do we “sell” family therapy? How do we win over family members to engage in a treatment that puts responsibility for change on everyone? In attachment‐based family therapy (ABFT), the relational reframe can help with this challenge.

Reframing is an intervention strategy used in many schools of therapy. “The meaning that any event has depends upon the ‘frame’ in which we perceive it. When we change the frame, we change the meaning” (Bandler and Grinder 1975, 5). Both cognitive‐behavioral and psychodynamic therapists help clients expand or alter their views or attributions about a problem to see it from a new perspective (e.g., Throop 2013). Family therapists use reframing to shift the focus from intrapsychic conflict to interpersonal conflict. The reframe has typically focused on shifting the perspective of “the youth as the problem” to “family relationships as the problem” (Nichols and Schwartz 2009). This legacy goes back to the 1940s, when Leo Kanner, a psychiatrist at Johns Hopkins, proposed that autism and schizophrenia were partially the results of the “refrigerator mother” (Eisenberg and Kanner 1956, 10). Unfortunately, this understanding created a reputation in the mental health field that family therapy still suffers from: we are the parent‐blaming therapists. Family therapist do walk a thin line between offering a systemic frame and conveying that “the child is not the problem, the parents are the problem.”

The concept of reframing has a long history in family therapy (Newsome et al. 2019). Minuchin (1974) defined reframing as redefining the symptoms or behavior (often referred to as relabeling) to create new possibilities for change or to enhance clients' emotional response. Haley and Richeport‐Haley (2004) referred to reframing as helping the clients move beyond hopelessness to feeling some agency about promoting change. Similarly, Watzlawick et al. (1974) considered reframing as a process that changes the way individuals view and experience a situation, both cognitively and emotionally. Thus, family therapists use reframing to shift the attribution or explanatory model about a person's behavior: “He is not running away from home because he hates you. He is running away because he feels like a burden to you.” Additionally, reframing might be used to shift family members' focus from individual functioning to contextual circumstances: “I know your daughter seems depressed, but I wonder if everyone is feeling depressed since the divorce.”

Given the centrality of reframing in family therapy, there is surprisingly little research to evaluate and understand this therapeutic process. Still, some interesting studies have been conducted. For example, Robbins et al. (1996) found that of all the therapist's statements, only reframing of adolescent negative behavior resulted in positive responses from the adolescent. Moran et al. (2005) found that when the therapist used relational reframes in the first session, parents were more likely to describe problems based on relational circumstance rather than child factors. In a study on unresolved anger toward attachment figures, an ABFT relational reframe intervention followed by an empty‐chair intervention sequence increased adaptive sadness and decreased maladaptive anger (Tsvieli et al. 2020). Hogue et al. (2019) looked for common intervention techniques across three structurally based, empirically supported family system treatments for delinquency and substance‐abusing youth. Reframing was one of the four primary intervention strategies found in all three models. These studies suggest that the reframe can and should be investigated more.

Attachment‐based family therapy (ABFT) uses the reframe in a slightly different way than some family therapy models. ABFT emerged in 2014 as an alternative to behaviorally focused family therapies and focuses more on psychological growth, emotional processing, relational trauma, and improving interactions that promote secure attachment. ABFT integrates attachment theory (Bowlby 1969) and emotional processing theory (Greenberg 2010) into a structural family therapy foundation. This empirically supported model has been effective with adolescents struggling with depression, suicide, trauma, and bulimia as well as with younger children and young adults (see review in Diamond et al. 2021). Rather than focus on day‐to‐day behavioral problems, ABFT aims to repair parent–child relational ruptures that have damaged the secure base of family life. Ruptures might result from family processes such as high conflict and low warmth or from family events such as loss, abuse, abandonment, and neglect. Addressing and repairing these ruptures can restore attachment security and its many developmental benefits.

The reframe is the primary focus of the first ABFT session. Rather than an occasional intervention or technique, the reframe sets the foundation of the therapy goals. The reframe shifts the goal from “fixing the adolescent” to “repairing family relationships.” Therapists promote the idea that repairing ruptures and improving felt security will reduce conflict. “I think if you and your daughter felt more connected, more trusting of each other, then some of these day to day conflicts might decrease.*”* This serves the traditional reframe function of shifting from the individual to the relational but also helps avoid the potential to blame the parents. Based on attachment theory, ABFT engages parents as the curative agent of change: “Mom and Dad, you are not the problem; you are the medicine!” Love and connection become the treatment goals.

Attachment theory guides this reframing process (Bowlby 1969). When securely attached children feel threatened or distressed, they instinctively turn to parents for protection and support. Parents instinctively become protective, responsive, and emotionally available. The child has an inherent desire to be cared for and parents have an instinct to care (Bosmans et al. 2022, 2020). Extensive research suggests that children in a secure based family environment use parents to cope with life's adversity, show more emotional and cognitive resilience, and have better interpersonal relationships with peers and romantic partners throughout life (Mikulincer and Shaver 2020).

The relational reframe intervention (or Task) has four well‐defined phases: joining, problem definition, reframing, and contracting (Table 1). The reframe phase (about 40 min into the session) begins with the therapist asking the adolescent, “When you feel so depressed or suicidal, why don't you go to your parents for help?” The conversation shifts from behavioral problems to focuses on family trust, safety, and support. In this phase, the therapist looks for processes (e.g., harsh criticism) or events (e.g., divorce) that have damaged trust. In this first session, the therapist does not dig deeply into these ruptures but finds enough information to highlight the consequence of these conflicts: the lack of connection and cohesion between family members. A therapist might say, “Look, divorce can be complicated, and we can sort this out more later. But clearly, since the divorce, you two have stopped feeling close.” The therapist then aims to amplify the instinctual, yet forgotten or smothered, longing for a better relationship.

**TABLE 1:** Four phases of the relational reframe task.

Task phase	Therapist's marker	Process targets
Joining	Let's spend a little time chatting, so I can get to know you	
Problem definition	OK, so tell me why you're here today and how I can help you	
Reframing	When you're in your room thinking of hurting yourself, why don't you go to your parents?	Content: from behavioral control to ruptures in family safety Affect: from anger and indifference to longing and love
Contracting	So how would you like if we initially focused this therapy on repairing or strengthening the relationships in the family?	Content: from behavior management to relationships repair Affect: from anger and indifference to hopefulness

The reframe uses several “levers” to help shift the goal of therapy (see Table 2). At the content level, we shift the conversation focus from behavioral problems (e.g., choirs, peers, school) to family interpersonal problems (divorce, parental illness, loss of trust, etc.). At the affect level, we shift from rejecting emotions (e.g., anger, frustration, indifference) to more vulnerable emotions (e.g., love, longing, sadness etc.). When these shift in content and affect occur, the therapist starts the contracting phase. At the content level, therapists shift the treatment goal from “fixing the adolescent” to “repairing relational security.” At the affect level, therapists shifts despair and resignation toward hopelessness and optimism. The therapist might say: “Seems like the divorce has been hard on everyone, but I worry that you and your daughter don't feel close anymore… How would you feel if we focus this therapy on repairing that distance between the two of you?”

**TABLE 2:** Content and affect during the reframe and contract phases.

	Reframe content	Reframe affect	Contract content	Contract affect
Indicators	Identify and discuss attachment ruptures	Express vulnerable emotions like longing or sadness	Accept relational repair as the focus of therapy	Express or display some Hopefulness that the relationships could improve
Scoring	0–3	0–4	0–3	0–4

### 1.1 Study Aims

The three aims of this study intend to dismantle, operationalize, measure, and test some of the core elements of the Reframing intervention. In Aim 1, we tested whether we could operationalize and code the Reframing and Contracting phases of the overall reframe intervention. Then, we aimed to operationalize and reliably code the intervention targets related to affect and content within each of these two phases. We coded to what extent the family members engaged in (reframe phase) or accepted (contract phase) the targets of each phase. In Aim 2, we explored if engaging in the reframing process was associated with accepting relationship repair goals (contracting). In Aim 3, we explored whether patient or parent baseline characteristics predicted how patients would respond to the interventions. We explored three potential moderators. First, we looked at whether adolescent attachment state of mind, measured with the adult attachment interview (AAI; George et al. 1985), might affect the reframing process. Focusing on adolescent attachment representations has particular relevance for the present study. Securely attached adolescents have confident expectations in their parents' availability and responsiveness, whereas insecure adolescents anticipate rejecting or inconsistent responses. These expectations, in turn, organize different strategies for maintaining the relationship when the adolescent is distressed (Kobak and Bosmans 2019). Secure adolescents are more likely to signal distress and approach parents for support.

Dismissing adolescents are more likely to shift attention away from parents and minimize distress when in distress. Preoccupied adolescents maximize signals of distress but are less likely to be comforted by contact with caregivers (Cassidy 1994). As a result, one could expect dismissing adolescents to resist the discussion of parent–adolescent relationship ruptures (reframe). In contrast, preoccupied adolescents may resist the offer of attachment repair (contract), with anger and resistance (Cassidy 1994). These complicated attachment‐informed responses to the intervention may complicate the therapist's attempts to steer family members to softer, more vulnerable emotions (Kobak and Bosmans 2019).

Further, we looked at parent‐ and adolescent‐reported family conflict and parent‐reported parental depression, two common factors associated with child distress and poor treatment response (Engelhard et al. 2022). We assumed that high family conflict might interfere with the therapist's attempts to uncover more vulnerable emotions and longing for attachment. We assumed that parental depression might dampen parents' emotional attunement to adolescents' needs (Joormann and Stanton 2016), thus inhibiting the acceptance of the relational repair goals of the reframe.

## 2 Method

### 2.1 Participants

The sample consisted of video recordings of ABFT therapy sessions from a National Institute of Mental Health‐funded randomized clinical trial comparing ABFT to a nondirective individual therapy for the treatment of adolescent depression and suicide (Diamond et al. 2019). At the time of this sub study, 47 ABFT cases were available. Adolescents' age ranged from 12 to 18 (*M* = 14.89; SD = 1.66). Eighty‐three percent were girls, 40% were Caucasian, 9.1% were Hispanic, and 16 (34.0%) had previously attempted suicide. Family income ranged between $5000 and $105,000 (*M* = $53,963; SD = $35,153). The relational reframe session occurred with the adolescent and the mother (*n* = 26), father (*n* = 4), two parents (*n* = 13), father and a stepmother (*n* = 2), mother and a stepfather (*n* = 1), or a grandmother (*n* = 1). All therapists had at least a master's degree and were trained in, and adhered to, ABFT procedures (Ibrahim et al. 2022). At least one parent (or main caregiver) participated with the adolescent in the treatment program. All participants provided written informed consent.

### 2.2 Measurement

#### 2.2.1 Relational Reframing Coding System

Sometimes process researchers can identify well‐developed process measures from other studies (e.g., emotional softening) to use in new studies. Other times investigators develop measures that capture model‐specific features. Using the latter approach, we developed the relational reframing coding system. In this, we operationalized the four phases of the relational reframe task: joining, problem definition, relational reframing, and relational contract. Then we operationalized the process targets of content and affect within the last two phases. These elements are captured in Table 1. Defining these units of study (phases) and process targets (affect and content) is a common step in psychotherapy change process research (Greenberg 1986).

#### 2.2.2 Coding Procedures

##### 2.2.2.1 Coding Phases

Two psychology undergraduate students without therapy experience were trained by the first author using 28 ABFT tapes from a prior study (Diamond et al. 2014). Students then coded the relational reframe (all first sessions) of ABFT. Coders identified the marker that began each phase. Each rater coded each tape twice: once focusing on the adolescent and once focusing on the parent.

#### 2.2.3 Coding Affect and Content

Once we could identify and isolate the phases, we developed codes to evaluate key process elements within the last two phases: content and affect. For these targets, we asked raters to judge whether, by the end of the phase, the adolescent and parent had engaged in exploration of the desired content and affect of the reframe and to what extent they seemed to agree to the relational repair focus of the therapy.

We made an adolescent and parent version of the codes to capture the subtle differences we expected (Table 2). All content scales were measured on a scale of 0 to 3 (0 = *refuses the discussion/denies the desire for attachment*; 3 = *clear acknowledgement and agreement that attachment is missing*). Affect scales were measured on a scale of 0 to 4 (0 = *inappropriate affect*; 3 = *clear and direct emotional engagement*; *resonating with emotional deepening*). The affect scores for the contract subscale ranged from negative affect (0) to clear positive affect, such as hope/relief/excitement (3).

We then coded videotapes to identify shifts in content and affect. We trained seven students to code reframe sessions with archived Task 1 tapes from a previous study. After training, we randomly divided all 47 Task 1 tapes from the Diamond et al. (2019) study among the student coders, with at least three students coding the same tape. The students observed each tape twice: once for coding the adolescent and once for coding the parents. We randomly alternated whether each student coded the adolescent or parent first so that there would be no ordering bias. In addition, when possible, there were different coders for the reframe and contracting phases for the same Task 1 tape. However, if the same student coder rated the reframe and contracting phases for the same tape, the coder always rated the contracting component first. Coders also had to write down their rationale for choosing a certain score. During the coding process, we met as a group four times to prevent rater drift by discussing complicated tapes and reaching a consensus.

#### 2.2.4 Adult Attachment Interview

We used AAI (George et al. 1985) to evaluate adolescents' states of mind with respect to attachment. Interviews were transcribed and sorted by raters using the 100 items AAI Q‐set (Kobak and Zajac 2011). Both raters had attended AAI coding workshops and passed reliability testing with Mary Main and Erik Hesse. Twenty‐one transcripts were coded by both raters who achieved 81% agreement on the three major categories Autonomous/Secure, Dismissing, and Preoccupied. Coders also sorted the 100‐item AAI Q‐set correlated with the Dismissing and Preoccupied prototypes. These correlations yield a dimensional index for Dismissing (*M* = 0.23; range −0.65 to 0.78) and Preoccupied states of mind (*M* = 0.03; range −0.55 to 0.72). The interrater correlations for the Dismissing and Preoccupied dimensions were 0.82 and 0.74, respectively. Dismissing strategies involve utilizing avoidant or diversionary strategies to shift attention away from attachment‐related vulnerabilities, whereas Preoccupied subjects are more likely to become enmeshed in interview topics (Hesse 2008). Adolescent security in the AAI is marked by a “Freedom to Evaluate” attachment topics and is evidenced by the adolescent's cooperative or coherent discourse with the interviewer's structured protocol of attachment topics and memories.

#### 2.2.5 Self‐Report of Family Functioning

Adolescents evaluated family conflict using the Self‐Report of Family Functioning (SRFF; Bloom 1985). There are 15 items in the SRFF, and it evaluates levels of family conflict, family cohesion, and democratic family styles. We used only the family conflict scores in this study. This scale consists of five items (e.g., “We fight a lot in our family.”), and participants respond from 1 (*never true*) to 4 (*very true*).

#### 2.2.6 Parental Depression

To evaluate parental depression, we used the Beck Depression Inventory‐II (BDI‐II; Beck et al. 1996), a 21‐question, 4‐point scale (from 0 to 3), where participants self‐report depressive symptoms in the recent 2 weeks. Questions involve psychological symptoms (e.g., helplessness, loss of interest) and physical symptoms (e.g., sleep changes, changes in appetite) in the past 2 weeks. The total self‐reported scores range from 0 to 63. Higher scores indicate more severe symptoms.

### 2.3 Procedures

We collected participants' baseline measurements (on the AAI, SRFF, and BDI‐II) at study intake for the RCT. Participants consented to having their therapy sessions videotaped. The study was approved by the Institutional Review Board of Drexel University.

### 2.4 Analysis

For Aim 1, we calculated interrater reliability with the intraclass correlation coefficient (ICC). For our analysis, we used the scores from two main coders, and we inputted the score of a third coder whenever we had missing scores.

For Aim 2, we tested whether the therapists' success in eliciting the targeted content and affect during the reframe phase would result in the family being more responsive and accepting of the targeted content and affect of the contract phase. We performed a Pearson correlation analysis between relational reframe (process) and contract components (outcome). We calculated 95% Confidence Intervals using a bootstrapping procedure (*k* = 5000 resamples). Both adolescents and parents' responsivity were analyzed and related to each other.

For Aim 3, we tested whether adolescent attachment, family conflict, and parental depression predicted the adolescent's and parents' responsiveness to the relational reframe behaviors during the reframing process and the contract outcome. To measure this, we performed a Pearson correlation analysis. We calculated 95% Confidence Intervals using a bootstrapping procedure (5000 bootstraps). We performed all statistical analyses with IBM SPSS Statistics version 27.

## 3 Results

### 3.1 Aim 1a: Interrater Reliability of the Relational Reframe Phases

The results showed that the different phases within the session could be reliably operationalized as independent phases of the therapy process. Results showed excellent ICC scores in the markers study, including joining (1.00), depression history (1.00), reframing (1.00), and contract (0.99). These values seem high but reflect that the relational reframe task in ABFT is clearly structured and offers a framework for task delivery.

### 3.2 Aim 1b: Coding Content and Affect for the Reframe and Contract Phases

We assessed the reliability of the affect and content codes by calculating inter‐observer agreement for 30 tapes that were double coded. Results indicated high ICC scores for all adolescent and parent scales (0.83 ≤ ICC(2,2) ≤ 0.99; see Table 3), with the exception of moderate ICC scores for affect scores during the contract component for mothers (ICC(2,2) = 0.69) and fathers (ICC(2,2) = 0.53). These results suggest that family‐member responsiveness during critical task components can be operationalized and reliably measured.

**TABLE 3:** Intraclass correlation coefficients of the Task 1 coding instrument.

Group	Reframe content	Reframe affect	Contract content	Contract affect
Adolescents	0.86	0.86	0.88	0.92
Mothers	0.92	0.87	0.87	0.69
Fathers	0.89	0.83	0.99	0.53

### 3.3 Aim 2: Associations Between the Reframe Phase and the Contract Phase

**FIGURE 1:**
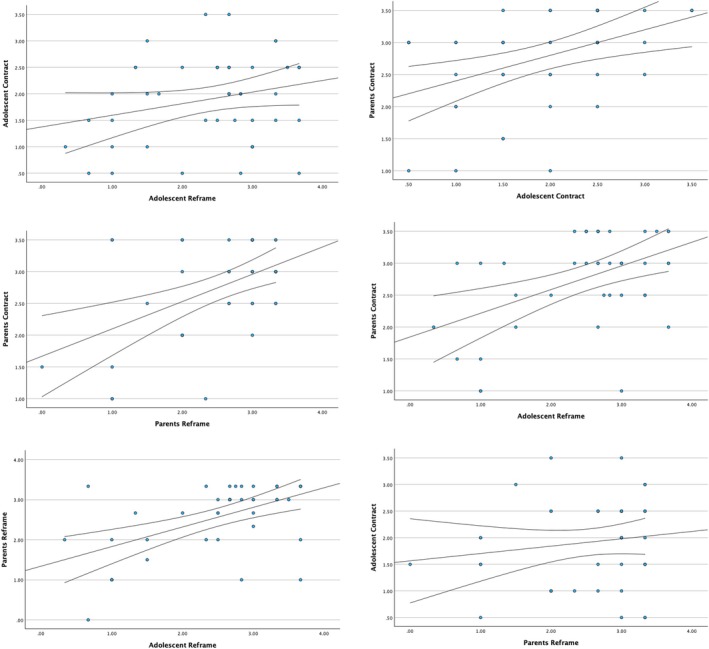
Scatterplots of the correlations between parents' and adolescents' reframe and contract scores.

Correlation analyses showed substantial overlap between the scales (with correlations up to *r* = 0.70 for adolescent process and contract). Moreover, factor analytic results supported a more parsimonious model in which the scales were combined. This decision reduced the number of statistical tests and therefore increased the conservativeness of hypothesis testing. For this purpose, we combined content and affect scores into one total score within the reframe and contract phases. We did this separately for the adolescents and parents. The Pearson correlations of parents' responses to the reframe process and contract were positive and statistically significant (Table 4; for visual inspection see Figure 1). The adolescent reframe and contract scores were not correlated (*B* = 0.22; SE = 0.13; 95% CI = −0.04 < *B* < 0.43). Parental reframe and contract scores were correlated (*B* = 0.43; SE = 0.12; 95% CI = 0.11 < *B* < 0.70). When parents had higher total scores in the reframing phase, they were more likely to have higher total scores on the contracting phases. Adolescent scores in the reframing phase were associated with the parent scores in the reframing phase (*B* = 0.49; SE = 0.12; 95% CI = 0.16 < *B* < 0.80) with higher scores for adolescents being linked to higher scores for parents. Similarly, adolescent scores on the contracting phase were associated with parents' contracting phase scores (*B* = 0.40; SE = 0.12; 95% CI = 0.16 < *B* < 0.66). Moreover, when adolescents had higher scores on the reframe phase (e.g., more focus on attachment ruptures and expression of softer emotions), parents were more likely to have higher scores in the contracting phase (accept the relational goals of the therapy; *B* = 0.37; SE = 0.11; 95% CI = 0.13 < *B* < 0.60). Finally, parental reframe phase scores were not related to adolescent contracting scores (*B* = 0.14; SE = 0.15; 95% CI = −0.13 < *B* < 0.39).

**TABLE 4:** Pearson correlations between adolescents' and parents' relational reframe and contract.

		Adolescents		Parents	
		Reframe	Contract	Reframe	Contract
Adolescents	Reframe	1	—	—	—
	Contract	0.25	1	—	—
Parents	Reframe	0.53**	0.15	1	
	Contract	0.47**	0.43**	0.50**	1

*Note:* **p < 0.01 (2‐tailed).

### 3.4 Aim 3: Baseline Predictors of the Reframe Contract Phases

Adolescents with dismissing attachment representations were not significantly more likely to have lower scores on the reframing phase, although the unstandardized regression value fell within the boundaries of the bootstrapped 95% CI (*B* = −0.57; SE = 0.30; 95% CI = −1.18 < *B* < −0.01). Moreover, a dismissive style was negatively, significantly, associated with adolescents accepting the relational contract (*B* = −0.82; SE = 0.24; 95% CI = −1.25 < *B* < −0.42; Table 5). In contrast, preoccupied attachment was linked to adolescents' increased engagement in the reframing phase (shift in content and affect; *B* = 0.83; SE = 0.37; 95% CI = 0.29 < *B* < 1.41) but was not related to acceptance of the relational contract (*B* = 0.49; SE = 0.12; 95% CI = 0.16 < *B* < 0.80). Adolescent attachment style was not related to the parents' response to the reframing or contracting processes.

**TABLE 5:** Pearson correlations between attachment, family conflict, and parental depressive and the response to the reframing and contract interventions.

	Adolescents		Parents	
	Reframe	Contract	Reframe	Contract
Dismissing attachment	−0.27	−0.45**	0.01	−0.30
Preoccupied attachment	0.32*	−0.03	0.16	0.00
Family conflict	0.03	−0.42**	−0.04	−0.06
Parental depressive symptoms	0.25	0.10	0.22	0.22

*Note:* *p < 0.05 (2‐tailed); **p < 0.01 (2‐tailed).

Adolescent‐rated family conflict was not related to the acceptance of the reframing phase but negatively related to adolescents' acceptance of the contract phase (*B* = −0.50; SE = 0.16; 95% CI = −0.73 < *B* < −0.26). The higher the conflict, the less likely the adolescent was to accept the relational focus of therapy (Table 5). No link was found with parents' scores on reframing or relational contracting. Finally, there were no significant correlations between parental depression symptoms and adolescent or parent responses to the reframe or contracting phases. However, for the analysis including the adolescents' reframe scores, the unstandardized regression value fell within the boundaries of the bootstrapped 95% CI (*B* = 0.44; SE = 0.26; 95% CI = 0.01 < *B* < 0.92). This suggested that adolescents with more depressed parents respond better to the relational reframe phase of the task.

## 4 Discussion

In this study, we explored the process and outcome of the Relational Reframe task, the initial session of ABFT. First, we dismantled the intervention into four phases. Then we identified whether the intervention resulted in the targeted shifts in content and affect assumed necessary for intervention success. Success of the intervention was defined as a high degree of acceptance of the relational contract: improving relationships rather than controlling behavior. To measure all this, we created a novel coding instrument to assess adolescents' and parents' responsiveness to the reframe and contract phases of the relational reframe task. Furthermore, we explored potential predictors of parent and adolescent responsiveness to this task, including adolescents' attachment representations, family conflict, and parental depressive symptoms.

Results suggested we could reliably identify the four phases of ABFT's relational reframe task. We could also reliably measure content and affect responses of the adolescent and parents to each relational reframe component. Adolescents' engagement in the reframe phase and acceptance of the relational contract was linked to parents' engagement and acceptance of the process and contract. Moreover, adolescents' attachment quality and perceived family conflict predicted adolescents' responsiveness during the relational reframe task.

### 4.1 Aim 1a and 1b: Interrater Reliability of the Relational Reframe Task Coding System

First, we found we could reliably operationalize and code for the four phases of the relational reframe task (Aim 1a). Operationalizing these therapy elements was essential for studying the structure and mechanism of the task. These findings also suggest that although psychotherapy can be an elusive process, it is possible to find repetitive structures that capture the core elements of a therapeutic moment. We encourage other investigators and even therapists to think about the common elements of their therapeutic strategies as a means to intensify, monitor, repeat, and teach what they do.

For Aim 1b, we found we could reliably operationalize two of the process targets (affect and content) within the last two phases of the task. For most scales, the ICCs were high, suggesting that it is feasible to identify how parents and adolescents respond to these phases of the task. Coders struggled more to reliably assess parents' affect during the relational contract. It might be that adult affective shifts during the relational contract are more complex and varied than those of adolescents. For example, adolescents are either mad or withdrawn. Parents, however, might be worried, scared, angry, frustrated, and sad all at the same time, making it difficult to clearly identify affective shifts. However, the results may also reflect a statistical artifact. Given the number of analyses performed, these lower values may reflect random variation rather than systematic reliability issues.

### 4.2 Aim 2: Associations Between the Reframe Phase and the Contract Phase

The data suggested that the association between engaging in the discussion of relational ruptures and accepting the relational treatment contract was not significant for adolescents. This finding suggests that even when adolescents respond poorly to the reframe (i.e., little shift in content and affect), they can remain open to participating in therapy focused on repairing their relationship with their parents (contract phase). Therefore, therapists should not give up too easily. Indeed, De Jonge et al. (2022) have shown that adolescents continue to desire parental support to help cope with life stressors. As a result, even when their attachment bonds get ruptured, adolescents retain a strong desire for their relationships with parents to be restored (Verhees et al. 2022). This seems to be reflected in their acceptance of the relational contract irrespective of the quality of the relational reframe process.

In contrast, parents who engaged more in the content and affect of the reframe process (a shift to ruptures and a softer emotion) were more likely to accept the relational contract. This finding highlights the importance of shifting content and deepening the emotion during the reframing for the parents. Acknowledging and amplifying parents' sadness or disappointment resulting from attachment ruptures with their adolescents seems an important precursor for parents to accept the relational contract. Moreover, parents' engagement in the reframe themes was also positively related to adolescents' engagement in the reframing process. This finding suggests that when adolescents soften and become more vulnerable, it activates parents' desire to care for their child, thus helping parents agree to the relational repair focus of the therapy. Moreover, the parents' acceptance of the relational contract was associated with the adolescents' acceptance of the relational contract. Again, when adolescents are engaged in the therapy process, parents become more receptive to the relational focus of the intervention.

### 4.3 Aim 3: Predictors of the Relational Reframe Process and Contract

We found that the three moderators (adolescent attachment orientation, adolescent‐reported family conflict, and parents' reported depressive symptoms) predicted adolescents' receptivity to the relational reframe process and acceptance of the relational contract. These moderators had no association with the parents engagement in the task. Adolescent dismissing states of mind were negatively linked to adolescents' responsiveness to the relational reframe and contract phases. Dismissing adolescents were more hesitant to discuss relational disappointments and less receptive to a therapy focused on relational repair. Dismissive youth tend to restrict their desires for love and attention and may be presented as defensive and indifferent, so this is not surprising (Kobak and Zajac 2011). However, this is not getting in the way of the therapy; it is the therapy. In Task II, alliance with the youth, we will focus more on their hurt, anger and hopelessness that fuels their relational indifference (i.e., protection).

Preoccupied attachment was also associated with treatment response. Interestingly, preoccupied youth more willingly engaged in the emotional process of discussing relational ruptures. However, they were more hesitant about the relational contract. Again, this is consistent with how we understand preoccupied attachment. These adolescents more easily express vulnerable emotions of hurt and disappointment (Verhees et al. 2021), which facilitates the relational reframe process. Typically, these adolescents crave support and understanding. However, they remain afraid of getting hurt again, which may contribute to the reluctance to the relational repair contract. Trying to understand, articulate, express, and work through the ambivalence about trusting parents again is the primary target work of the ABFT model.

The observation that adolescents' attachment was not linked to parents' response to the reframe process and the relational contract should be treated cautiously. The sample size was small and this could leave some associations undetected. However, if replicated, this could be considered a hopeful observation. Parents were equally likely to respond well to the relational reframe task regardless of the adolescent having a secure or insecure attachment style.

Higher adolescent‐reported family conflict increased adolescents' but not parents' resistance to the relational contract. Family conflict might increase adolescents' fear that parents will not change. Researchers have argued that family conflict masks the desire for closeness with high levels of fear for rejection (Kobak and Bosmans 2019). Thus, it is possible that adolescents from high conflict families still retain their fear and doubt when therapists invite them to work on getting closer to their parents (relational contract). The lack of an association between family conflict and parents' relational contract should be considered cautiously, as larger sample sizes could have detected small effects. Moreover, the different results could reflect reporter biases and even reporter discrepancies (e.g., Ehrlich et al. 2016). At this moment, scores suggest that family conflict is less of a factor in parents' acceptance of the relational contract. High family conflict is difficult to manage in the room but should not dissuade therapists from pursuing the goal of improving love and trust.

Finally, parental depression was initially not associated with any of the reframing constructs. However, after bootstrapping, the association between parental depression and adolescents' response to the reframe phase appeared significant: parental depression increased adolescents' positive response to the discussion or ruptures and expression of vulnerable emotions. This may reflect that adolescents of depressed parents usually do not want to burden their parents, thus inhibiting their emotional needs. The reframe process gives them permission to express avoided vulnerable emotions. Couple this with our finding that adolescent engagement in the task improves parental engagement in the task, and adolescent engagement may help buffer the tendency for depression to heighten parental resistance to treatment (Ofonedu et al. 2017). More research is needed to see whether this effect is replicated and to investigate what it might mean. Moreover, similar research should be conducted for different parent psychopathology indices, but the present study suggests that at least for initiating ABFT, parents' depressive symptoms are not a direct deterrent to the treatment goals.

## 5 Limitations

Several limitations should be acknowledged. First, we focused only on the first session of ABFT, the relational reframe task. In practice, we know that in ABFT the development of relational themes and engagement in relational repair evolves over several sessions; it remains the focus of the first four tasks. The first session is only the beginning of this conversation. We also only looked at proximal outcomes at the end of the first session. In another paper, we are exploring how engagement in the reframe and acceptance of the contract might be predictive of treatment outcome (Bergers et al. 2021). Nevertheless, dissecting and studying this complex initial intervention step provides meaningful insights into processes that the therapist can use to help think about the intervention. Second, we tested only a few pretherapy variables (i.e., the adolescents' attachment style, family conflict, parental depressive symptoms). Other variables may be more predictive of families who engage or resist the relational reframe. Third, we developed a new coding system for this project, rather than using well‐validated tools that could measure well established psychotherapy processes. We could have used emotional processing tools or alliance measures. However, we wanted a tool that got close to the specific clinical phenomena that represent the proposed model of change. We also could have chosen a tool that focuses on the interaction between family members, rather than just individual processes. However, in the therapy we are tracking individual responses to help select intervention strategies that facilitated better interactions. We were pleased that our measures had good interrater reliability, suggesting that the constructs of interest were well operationalized.

Finally, we are aware that small sample size studies like this may limit what we can find. Furthermore, most of the analyses were correlational, which does not imply causality. We discuss it as causality because of the temporal sequence of the phases: reframing precedes contracting. Clinical intuition suggests that what we do first affects what we do next.

## 6 Implications and Future Directions

The relational reframe task encourages therapists to work faster and deeper. In the first session, we focus conversations on the relational ruptures that have undermined trust and cooperation. Although the road map of Task I is clear, the idiosyncratic responses of family members complicate the delivery of the session. ABFT encourages therapists to have a clear intention about the goal of the therapy but then use the family's story and experience to achieve this goal. The resistance to the relational goal is not getting in the way of the therapy; it is the therapy. The question of what gets in the way of trust and love organizes the entire treatment course. It appears from this data that the parents' early response to this focus is not dependent on the adolescents' response but is enhanced when the adolescent responds positively.

Further, our analyses show that adolescents' insecure attachment informs adolescents' responses to the relational reframe process and contract. This insight encourages therapists to provide more sensitive support for the adolescents' attachment fears and lend them hope and motivation for relational repair. Interestingly, we did not find family conflict and parental depressive symptoms to inhibit engagement in reframing and contracting. This helps assuage therapists that family therapy in general, and ABFT in particular, will be more difficult in high conflict families or families with a depressed parent.

## Funding

This work was supported by the National Institute of Mental Health, NCT01537419.

## Conflicts of Interest

Dr. Satens is an employee, and Dr. Bosman is the director of the attachment‐based family therapy training center at Luven. Drs. Diamond and Levy are funded by the ABFT International Training Institute in Philadelphia. They also received royalties from the ABFT treatment manual and honorarium for giving private talks on ABFT.

## Data Availability

The data that support the findings of this study are available from the corresponding author upon reasonable request.
